# An olfactory demography of a diverse metropolitan population

**DOI:** 10.1186/1471-2202-13-122

**Published:** 2012-10-10

**Authors:** Andreas Keller, Margaret Hempstead, Iran A Gomez, Avery N Gilbert, Leslie B Vosshall

**Affiliations:** 1 Laboratory of Neurogenetics and Behaviour, The Rockefeller University, 1230 York Avenue, Box 63, New York, NY, 10065, USA; 2 Howard Hughes Medical Institute, The Rockefeller University, 1230 York Avenue, Box 63, New York, NY, 10065, USA; 3 Synesthetics, Inc, Montclair, NJ, 07043, USA; 4 Present address: St. Vincent Medical Center, Bridgeport, CT, 06606, USA

**Keywords:** Olfaction, Psychophysics, Demographics

## Abstract

**Background:**

Human perception of the odour environment is highly variable. People vary both in their general olfactory acuity as well as in if and how they perceive specific odours. In recent years, it has been shown that genetic differences contribute to variability in both general olfactory acuity and the perception of specific odours. Odour perception also depends on other factors such as age and gender. Here we investigate the influence of these factors on both general olfactory acuity and on the perception of 66 structurally and perceptually different odours in a diverse subject population.

**Results:**

We carried out a large human olfactory psychophysics study of 391 adult subjects in metropolitan New York City, an ethnically and culturally diverse North American metropolis. 210 of the subjects were women and the median age was 34.6 years (range 19–75). We recorded ~2,300 data points per subject to obtain a comprehensive perceptual phenotype, comprising multiple perceptual measures of 66 diverse odours. We show that general olfactory acuity correlates with gender, age, race, smoking habits, and body type. Young, female, non-smoking subjects had the highest average olfactory acuity. Deviations from normal body type in either direction were associated with decreased olfactory acuity. Beyond these factors we also show that, surprisingly, there are many odour-specific influences of race, age, and gender on olfactory perception. We show over 100 instances in which the intensity or pleasantness perception of an odour is significantly different between two demographic groups.

**Conclusions:**

These data provide a comprehensive snapshot of the olfactory sense of a diverse population. Olfactory acuity in the population is most strongly influenced by age, followed by gender. We also show a large number of diverse correlations between demographic factors and the perception of individual odours that may reflect genetic differences as well as different prior experiences with these odours between demographic groups.

## Background

Compared to other senses, olfactory perception is considered to be variable, subjective, and unreliable. Both variability between individuals and within individuals contribute to the overall variability. The same olfactory stimulus can be perceived differently by the same subject on different occasions and, as a consequence, olfactory psychophysics has unusually high within-individual variability [1], which can be of the same magnitude as variability between individuals [2]. However, careful psychophysical experiments have also revealed large, stable inter-individual differences in olfactory perception. There are dramatic differences in subjects’ sensitivity to odours [3,4] and the perceived quality and pleasantness of some odours differs greatly between individuals [5,6]. The inter-individual differences can be general [3] or they can concern specific odours [4,7].

Many factors contribute to inter-individual differences in general olfactory acuity, which is a measure of olfactory abilities across different stimuli. A reduced general olfactory acuity can be genetic [8-10] or it can be caused by trauma [11], exposure to toxic agents [12], neurodegenerative diseases [13], or infections [14]. General olfactory acuity declines with age [15,16] and is lower in men [17,18]. In contrast, odour-specific inter-individual differences have only been shown to be influenced by variability in the gene for an odorant receptor that is responsive to the odour [19-23] and by previous experience with the odour in question [24-27].

**Figure 1:**
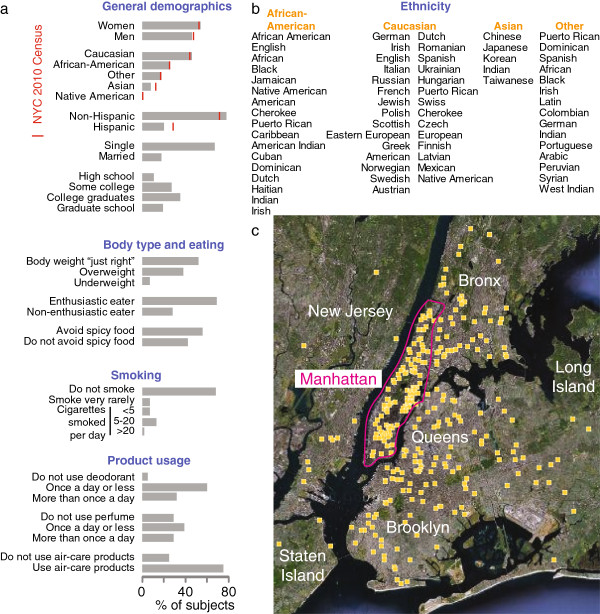
**Subject population. a**, Self-reported demographic, personal habit, and product usage information about the subject population compared (when available) to 2010 US Census data for New York City, as indicated by the red vertical line. **b**, Self-reported ethnicity of the subject population sorted by self-reported race and listed with the most frequently reported ethnicities at the top. Data for Caucasians are split into two columns, such that “German” is the most frequently and “Native American” the least frequently reported ethnicity. Subjects could self-identify as more than one ethnicity and only ethnicities that were used by at least two subjects are shown. 75% of “Other” subjects self-;identified as Hispanic compared to fewer than 10% in the three main racial groups. **c**, Primary residence of 93% of study subjects is indicated as a yellow square on a satellite map of the NYC metropolitan area, labelled to indicate the five boroughs, New Jersey, and Long Island. The approximate boundary of Manhattan is indicated by the magenta line. In some cases many subjects were from the same area and their individual locations cannot be resolved here. The remaining subjects did not have their primary residence within the area covered by the map.

Here we explore all these aspects of variability in a dataset that was collected to identify genetic variations in odorant receptor genes that influence the perception of specific odours [19]. This dataset, which is made available here in its entirety for data-mining purposes (Additional file 1), has three features that make it exceptionally useful for the study of perceptual variability. First, the same psychophysical measures were collected from each subject on two visits on two different days. This allowed us to quantify the relative contributions of inter-individual variability and within-individual variability to overall variability and to control for the latter when desired. To further investigate within-individual variability, 56 of the subjects returned for a third and fourth visit more than one year after the second visit. Second, our subject population was unusually diverse and closely reflected the age structure and ethnicity of New York City (Figure 1) [28]. This is an advantage over typical psychophysical or psychological studies that rely on the comparatively homogeneous subject pool of college students [29,30]. Third, we have assessed responses to a large panel of diverse odours. This is a marked difference from typical studies of olfactory perception, which often focus on a limited number of odours. Consequently, we have uncovered a large number of odour-specific differences between demographic groups that will inform the study of genetic and environmental influences on olfactory perception.

## Results and discussion

### Within-individual variability

**Figure 2:**
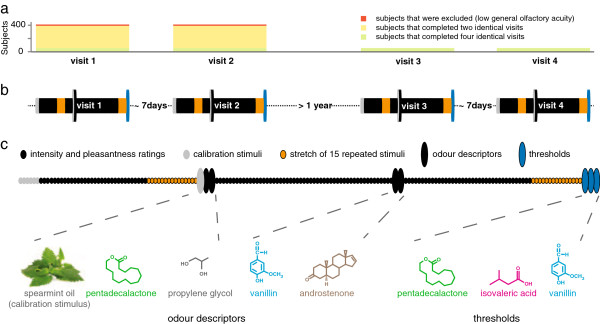
**Experimental design.a**, The study involved four identical visits. 412 subjects completed the first two visits. Of those, the 21 with the lowest general olfactory acuity were excluded (subjects in red). 56 subjects were invited for visits three and four (subjects in green). **b**, There was a spacing of approximately seven days between visits one and two and between visits three and four. There was a spacing of more than one year between visit two and three. **c**, During each visit, the intensity and pleasantness of 159 stimuli was rated. After the subjects rated 53 stimuli they assigned descriptors to spearmint oil, pentadecalactone, and the solvent propylene glycol. After they rated 106 stimuli, they assigned descriptors to vanillin and androstenone. After the intensity and pleasantness rating the thresholds for pentadecalactone, isovaleric acid, and vanillin were measured. See the “odours and sequence of stimuli” tab of Additional file 1 for the complete sequence of odours and concentrations used in the study.

**Figure 3:**
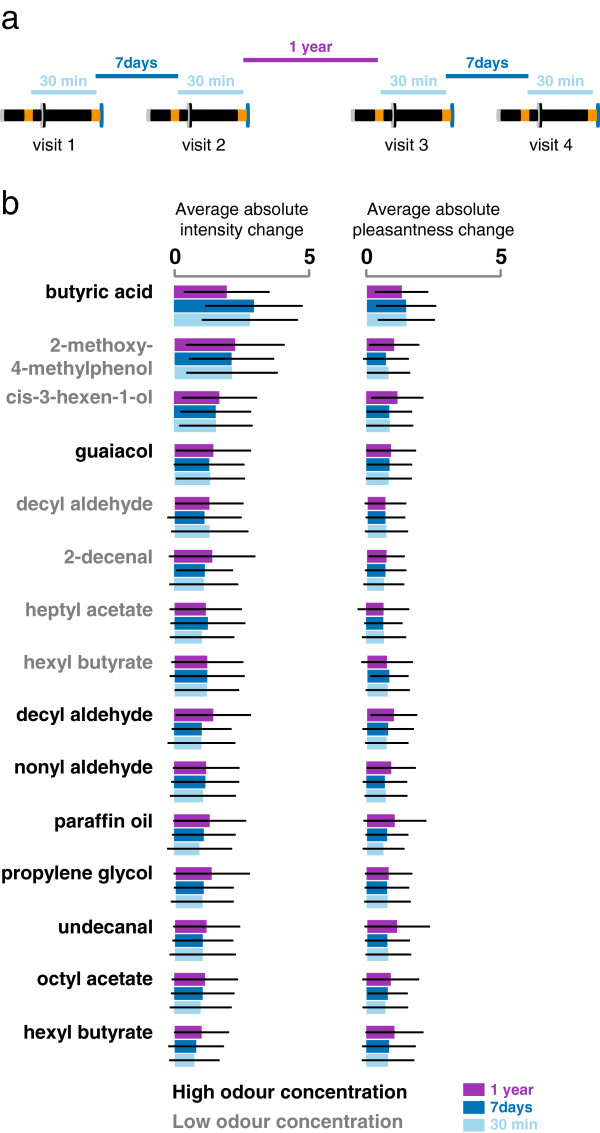
**Within**-**individual variability of odour perception.** The average absolute change in intensity and pleasantness ratings of fifteen stimuli were measured for 56 subjects twice on each of four visits. **a**, The absolute intensity and pleasantness changes were calculated by averaging the absolute difference between two ratings performed by the same subject. For the 30 min measure, the first and second ratings were compared for all four visits. For the 7 days measure, the second rating of the first visit and the first rating of the second visit and the second rating of the third and first rating of the fourth visit were compared. For the 1 year measure, the second rating of the second visit and first rating of the third visit were compared. There was about one week between visits one and two and between visits three and four. Visits two and three were more than one year apart. **b**, The average absolute change in intensity (left) and pleasantness (right) ratings for each stimulus is shown for three time intervals between ratings: 30 minutes, one week, and more than one year (519 days on average). An absolute change of “1” is equivalent to a change of one step on the rating scale (for example from “very weak” to “extremely weak”). Data are plotted as mean±standard deviation of the mean (S.D.). Odour names are labelled according to odour concentration: grey: low; black: high.

To quantify within-individual variability over time, 56 of the 391 subjects rated the intensity and pleasantness of 15 stimuli presented at intervals ranging from 30 minutes to over one year. On each of four visits, these subjects rated the 15 stimuli twice, approximately 30 minutes apart. The second visit was on average 7 days after the first visit and the third visit was on average 519 days after the first visit. The fourth visit was on average 7 days after the third visit (Figure 2). The within-individual variability (Figure 3a) was higher for the intensity ratings (Figure 3b; left) than for the pleasantness ratings (Figure 3b; right; p<0.0001). Furthermore, within-individual variability was odour-dependent. The variability of the perceived intensity of the high concentration of butyric acid (Figure 3b; top), for example, was significantly higher than the variability of the intensity perception of the high concentration of hexyl butyrate (Figure 3b; bottom; p<0.0001). Interestingly, for any given stimulus, the responses were as similar when the ratings were spaced over one year apart (Figure 3b; dark blue bars) as when they were around 30 minutes apart (Figure 3b; light blue bars). This may seem surprising, but for thresholds it has even been reported that the variability within a day is significantly larger than the variability between days [2]. Day-to-day variability in olfactory perception is therefore largely a consequence of sniff-to-sniff variability. The main causes of within-individual variability are processes that operate on the scale of seconds or minutes such as changes in the stimulus signal-to-noise ratio [31] or the reallocation of attention by the subject [32], rather than on the scale of hours or days, such as hormonal changes or infections of the upper respiratory tract.

### Variability in general olfactory acuity

For all remaining data evaluation, we minimized the influence of within-individual variability by averaging data from the two first visits. To study the influence of demographic factors on general olfactory acuity, we ranked our subjects according to their overall acuity, yielding a ranking of 391 (highest olfactory acuity in the study) to 1 (lowest olfactory acuity in the study). General olfactory acuity was composed of both thresholds and intensity ratings (see Methods for details). The measure of general olfactory acuity used here is not one of the standardized tests for olfactory acuity, such as the University of Pennsylvania Smell Identification Test (UPSIT) [33], the phenyl ethyl alcohol single staircase odour detection threshold test, or numerous others [1]. However, by averaging six performance indicators that are influenced by the perception of 66 structurally different odours, we obtain a uniquely comprehensive measure of general olfactory acuity.

**Figure 4:**
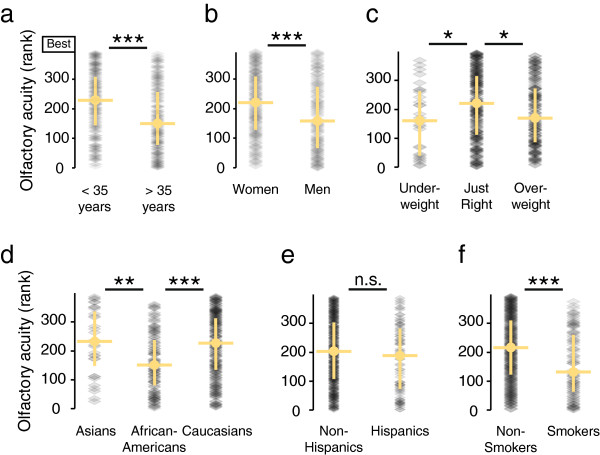
**General olfactory acuity.a**-**f**, Olfactory acuity ranks for different demographic groups are shown and compared. Each subject was assigned a rank between 1 and 391, with 391 signifying the highest, or best, olfactory acuity. Subjects are represented by grey dots and the median and first and third quartile of each group are shown. A two-tailed Mann–Whitney test (*p<0.05; **p<0.01; ***p<0.001; n.s.: not significant) was performed to test the differences for statistical significance (Ns: < 35 years: 195; > 35 years: 196; Women: 210; Men: 181; Underweight: 28; Just Right: 202; Overweight: 149; Asians: 31; African-Americans: 97; Caucasians: 178; Non-Hispanic: 305; Hispanic: 77; Non-Smokers: 290; Smokers: 92). Subjects were divided by age according to the median of 34.6 years, which is rounded to 35 years for labeling the figure.

Increased age had a large influence on general olfactory acuity in our population. Those above 34.6 years of age had a lower acuity (median rank: 152) than those below 34.6 years of age (median rank: 231). That olfactory acuity declines dramatically with age is well known [15,16,34,35] (Figure 4a). It is unclear if this decline with age is a part of the normal aging process or the cumulative effect of damage to the olfactory system caused by upper respiratory infections, trauma, or environmental toxins [11,12,14].

Another large influence on general olfactory acuity was gender. In our population, women had a more acute sense of smell (median rank: 220) than men (median rank: 159), which confirms previous reports [17,18] (Figure 4b). The difference in general olfactory acuity between the genders has been previously shown to be more pronounced in older subjects than in younger subjects [34,36], however we found no evidence for this in our dataset (data not shown).

We also found significant differences between self-reported body type and general olfactory acuity. Subjects who rated themselves either underweight (median rank: 161) or overweight (median rank: 171) had a lower general olfactory acuity than subjects who rated their body weight as “just right” (median rank: 219) (Figure 4c). It has been suggested that metabolic changes occurring in obese individuals have a negative influence on olfactory acuity [37]. However, in a recent study it was shown that the olfactory acuity of subjects who experienced dramatic weight loss after gastric bypass surgery did not improve [38]. The causality may therefore be in the other direction. Instead of obesity causing reduced olfactory acuity, olfactory dysfunction could be a contributing factor to the development of obesity. This effect could be mediated by the influence of olfactory function on food choice and food intake [39,40] and is deserving of further study.

Consistent with earlier reports on differences in olfactory perception between racial groups [33], there were differences in our measure of olfactory acuity between races (median ranks: African-Americans 149; Asians 231; Caucasians 225) (Figure 4d), but not between Hispanics (median rank: 186) and Non-Hispanics (median rank: 201) (Figure 4e). Other demographic factors such as marital status or education did not correlate with olfactory acuity (data not shown; see the “demographics” tab of Additional file 1 for tabulation of these demographic data).

The effect of smoking on the sense of smell is still the subject of debate [41]. In a study of 1,387 Swedish subjects, no statistically significant relationship between olfactory dysfunction and smoking was found [42]. Among 2,838 participants from the state of Wisconsin in the United States, smoking was associated with increased odds of olfactory impairment in women, but not in men [35]. Among 1,312 Germans tested, smokers had an impaired sense of smell [43]. An impaired sense of smell has also been observed in other studies [44,45], and our results also suggest that smokers (median rank: 130) have lower olfactory acuity than non-smokers (median rank: 213) (Figure 4f). Female smokers (median rank: 173) had a lower general olfactory acuity than female non-smokers (median rank: 233) and male smokers (median rank: 91) had a lower general olfactory acuity than male non-smokers (median rank: 190).

### Variability in sensitivity to specific odours

**Figure 5:**
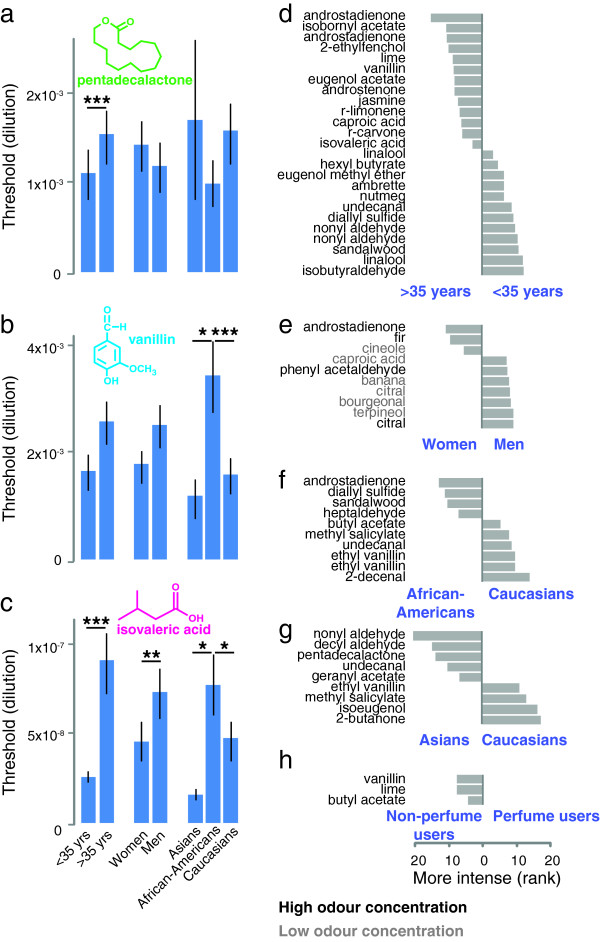
**Perception of odour intensity.ac**, Thresholds for pentadecalactone (**a**), vanillin (**b**), and isovaleric acid (**c**) for different demographic groups. A two-tailed Mann–Whitney test (*p<0.05; **p<0.01; ***p<0.001; n.s.: not significant) was performed to test the differences for statistical significance. Mean±S.D. are shown. **dh**, Differences in intensity rank of stimuli. Only statistically significant differences are shown. The test for statistical significance was a two-tailed Mann–Whitney test with a sequential Bonferroni correction called the Holm’s method to correct for multiple comparisons (p<0.0082) [47]. Odour names are labelled according to odour concentration: grey: low; black: high; (Ns: < 35 years: 195; > 35 years: 196; Women: 210; Men: 181; Asians: 31; African-Americans: 97; Caucasians: 178; Perfume users: 267; Non-perfume users: 112). Subjects were divided by age according to the median of 34.6 years, which is rounded to 35 years for labeling the figure.

We next investigated odour-specific differences between demographic groups. Previous work suggested that odour-specific perceptual differences can be caused by genetic polymorphisms [19,20] or by learning and experience [24,25,46]. We measured detection thresholds of the musky odour pentadecalactone, the vanilla odour vanillin, and the sweaty sock odour isovaleric acid (Figure 2; Figure 5a-c). Subjects also rated the intensity of 66 odours at two concentrations (Figure 2; Figure 5d-h).

Odour detection thresholds contributed to our measure of general olfactory acuity (see Methods). Accordingly, all statistically significant differences between thresholds (Figure 5a-c) are between demographic groups that also have different general olfactory acuity. However, differences in the sensitivity to a specific odour do not necessarily reflect differences in general olfactory acuity. Although men, who have a lower general olfactory acuity than women, are less sensitive to most odours that have been studied [17,18], males have been shown to be more sensitive to the odour bourgeonal, which has a floral lily-of-the-valley scent [48]. Similarly, African-Americans have been shown to have a higher threshold for isovaleric acid than Caucasians, but a lower threshold for pentadecalactone [49]. The same trend is seen in our data, although the differences are not statistically significant (Figure 5a-c). This illuminates the limitation of a general olfactory acuity score, 50% of which is based on the sensitivity to three odours. If there are differences in the tuning of the olfactory system between demographic groups, the general olfactory acuity score may reflect these differences in tuning rather than differences in acuity.

The intensity rating of 66 odours allowed us to quantify how much the perceived intensity of specific odours varied across subjects. The same subject rated the same stimuli during the two visits to be of similar intensity. The median of the Spearman correlation coefficient for within-individual comparisons was 0.66. In contrast, different subjects differed widely in their assessment of the intensity of the stimuli. The median Spearman correlation coefficient for all pairwise comparisons between subjects was only 0.36. To identify the factors contributing to this inter-individual variability, we assessed how the perception of different stimuli varied across the major demographic groups in our study. The three stimuli showing the greatest variability between subjects in intensity ratings were the high concentrations of androstenone [standard deviation (σ)=2.09] and androstadienone (σ=2.01), two odorous steroids found in human body secretions [50], and methanethiol (σ=2.02), a cabbage-like odour present in urine of people who have previously ingested asparagus [51]. Our discovery of the large variability of methanethiol intensity perception is interesting because others have reported variability in the perception of the smell of asparagus urine odour. Some individuals cannot perceive the characteristic asparagus urine odour and the percentage of these individuals has been reported to vary between 0% and 50% in different demographic populations [52-55]. Recently, it has been shown that the inability to smell asparagus urine odour is associated with a single nucleotide polymorphism within a 50-gene cluster of olfactory receptors [52,56,57].

We and others showed previously that the intensity perception of androstenone and androstadienone—the two most variably perceived stimuli in our study—is altered by genetic variation in the odorous steroid-sensitive odorant receptor OR7D4 [19,21,23]. Here we show in the same subject population that androstadienone was perceived to be stronger by older subjects (Figure 5d) and women (Figure 5e). We also show here that Caucasians perceived androstadienone to be a weaker odour than African-Americans (Figure 5f). This is consistent with the finding from the National Geographic Smell Survey, which found that African respondents were more sensitive to androstenone than American respondents. This difference is undoubtedly at least partially caused by the fact that the functional RT variant of OR7D4 is more common in African-Americans than in Caucasians [19].

Among the other statistically significant differences in intensity perception between demographic groups it is remarkable that Asians perceive each odour of the homologous series of nonyl aldehyde, decyl aldehyde, and undecanal to be stronger than Caucasians (Figure 5g). The difference in the intensity perception of the high concentration of nonyl aldehyde is the largest difference between demographic groups that we found. We have no mechanistic explanation for this interesting perceptual difference in Asians, but the observation bears further investigation. It is also worth noting that we found that men perceive bourgeonal to be more intense than women (Figure 5e). Our results confirm a previous study that showed that bourgeonal is the only known odour that men are more sensitive to than women [48]. Three odours were perceived as more intense by non-perfume users (Figure 5h).

### Variability in perceived pleasantness of specific odours

**Figure 6:**
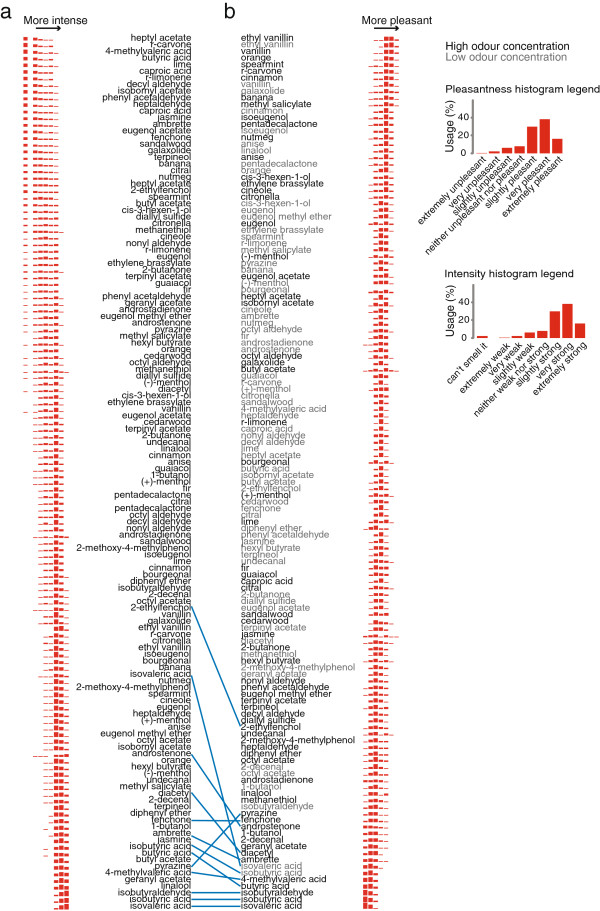
**Perception of odour intensity and pleasantness.a**-**b**, 66 odours at two concentrations are ordered according to how intense (**a**) or how pleasant (**b**) they are perceived to be by the subject population. Odour names are labelled according to odour concentration: grey: low; black: high. Histograms are shown to allow the evaluation of variability. The inset shows the legend for the histograms. The blue lines show statistically significant correlations between the perceived intensity and pleasantness of a given stimulus.

**Figure 7:**
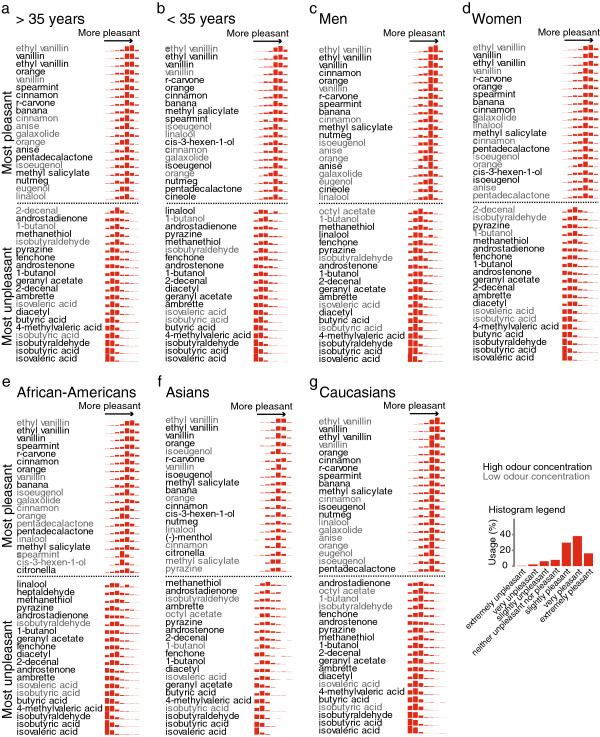
**Perception of odour pleasantness in different demographic groups.a**-**g**, The evaluation presented in **a**-**g** of seven demographic groups is derived from the full population in Figure 6. Only the 20 most pleasant and the 20 most unpleasant stimuli are shown for each group. Odour names are labelled according to odour concentration: grey: low; black: high. The inset shows the legend for the histograms (Ns: < 35 years: 195; > 35 years: 196; Women: 210; Men: 181; Asians: 31; African-Americans: 97; Caucasians: 178). Subjects were divided by age according to the median of 34.6 years, which is rounded to 35 years for labeling the figure.

The same 66 odours at two concentrations that were rated for intensity were also rated for pleasantness. In general, there was much stronger agreement in the subject population about the pleasantness of the stimuli (median Spearman correlation coefficient for all pairwise comparisons between subjects =0.68) than about their intensity (median Spearman correlation coefficient for all pairwise comparisons between subjects =0.36). However, perceived pleasantness also varies between individuals and it is known to depend on genetic variation [19], cultural background [27], and conditioning [58]. We found that intensity and pleasantness judgements were correlated, especially for unpleasant odours (blue lines in Figure 6), meaning that unpleasant odours were generally perceived to be more intense than pleasant odours. Across all subjects, the eight most pleasant odours were food odours such as vanilla, citrus, minty, and cinnamon odours, while the seven least pleasant odours were fatty acid derivatives associated with the sour smell of rancid butter or body odour (Figure 6b; most pleasant odour is displayed at the top). The biggest variability in pleasantness perception was found for floral odours [high concentrations of jasmine (σ=1.80), butyl acetate (σ=1.61), undecanal (σ=1.51), bourgeonal (σ=1.49), and eugenol (σ=1.49)] (Figure 6b). The two most pleasant stimuli were the two concentrations of ethyl vanillin, followed by the high concentration of vanillin (Figure 6b). Ethyl vanillin was rated to be more pleasant than vanillin by all demographic groups (Figure 7). The least liked odour was isovaleric acid (“sweaty socks”), followed by isobutyric acid (“rancid butter”), and isobutyraldehyde (“sour”) (Figure 6b). Throughout our subject population, odours perceived to be most and least pleasant were remarkably stable (Figure 6b).

**Figure 8:**
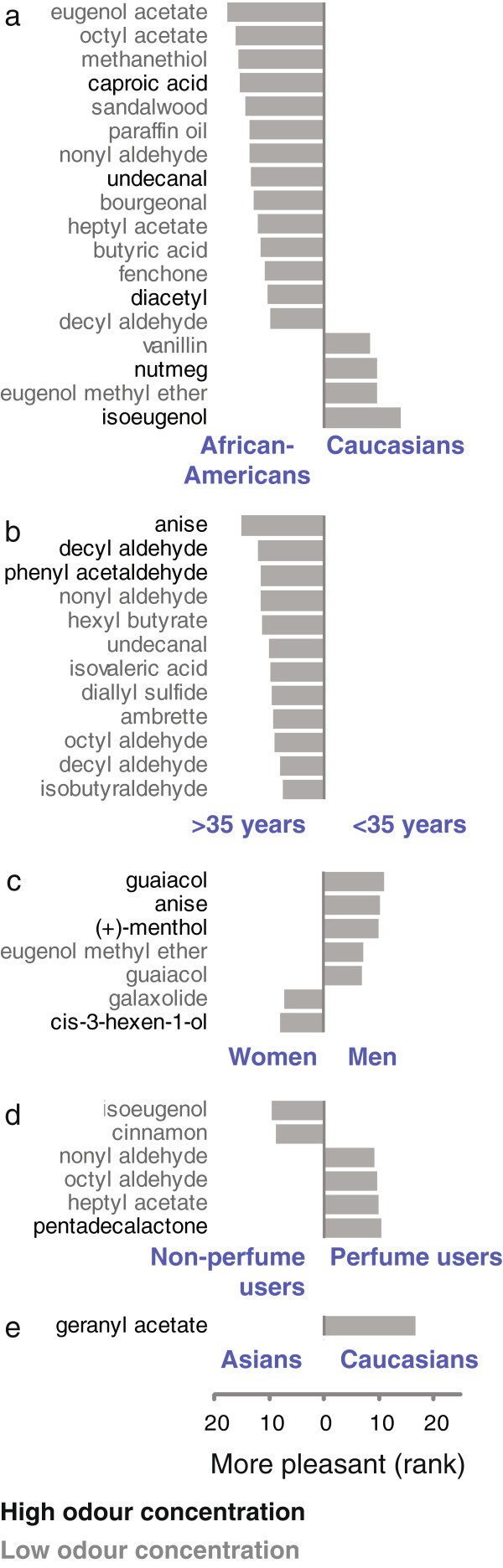
**Demographic differences in perceived odour pleasantness.ae**, Differences in pleasantness rank of stimuli. Only statistically significant differences are shown. The test for statistical significance was a two-tailed Mann–Whitney test with a sequential Bonferroni correction called the Holm’s method to correct for multiple comparisons (p<0.0082) [59]. Odour names are labelled according to odour concentration: grey: low; black: high; (Ns: < 35 years: 195; > 35 years: 196; Women: 210; Men: 181; Asians: 31; African-Americans: 97; Caucasians: 178; Perfume users: 267; Non-perfume users: 112). Subjects were divided by age according to the median of 34.6 years, which is rounded to 35 years for labeling the figure.

However, there were some dramatic differences between demographic groups. Statistically significant differences in pleasantness perception are shown in Figure 8. For 18 of the 134 stimuli the pleasantness rating differed significantly between African-American and Caucasian subjects (Figure 8a). The biggest difference between younger and older subjects was that older subjects perceived anise, the odour of liquorice, to be more pleasant (Figure 8b).

The first published differences in pleasantness perception of odours between women and men were described in 1924 [60]. In that study, women rated camphor, menthol, citronella, and ferric valerian as more pleasant than men, whereas men found cedarwood oil, pine oil, musk, and Tonka beans to be more pleasant [60]. A similar heterogeneity was found in the 1980s in the National Geographic Smell Survey, where amyl acetate and mercaptan were rated as more pleasant by men than by women, but rose and eugenol as more pleasant by women than men [61]. Furthermore, it was shown in a large study (N=301 subjects) that the odorous steroid androstenone is perceived to be less pleasant by women than by men [62]. The finding that women like androstenone less than men was not reproduced in our study. The difference in perceived pleasantness of eugenol, menthol, citronella, and cedarwood oil could also not be reproduced in our study. Instead, we found seven new stimuli whose pleasantness was perceived differently by men and women (Figure 8c). For guaiacol, the odour of wood smoke, both concentrations were perceived to be significantly more pleasant by men. The high concentration of guaiacol showed the largest difference between men and women (Figure 8c).

What accounts for these historical differences in gender-dependent pleasantness between our study and earlier studies? It may be that the perceived pleasantness of odours has a cultural component [27,63] and culture changes over time. Pleasantness perception is likely to be determined at least partially by cultural associations. We propose that the associations men and women had with odours in the 1920s and the 1980s are different from what they are in the 2000s.

Possibly because of the cultural component of odour pleasantness perception, all the odours that were perceived to be more pleasant by perfume users were odours used in perfumes (pentadecalactone, heptyl acetate, octyl aldehyde, nonyl aldehyde) (Figure 8d). Octyl aldehyde and nonyl aldehyde are key ingredients of perfumes, including the classic scent Chanel No. 5 [64]. Perfume use may result in these odours being rated as more pleasant, or, alternatively, those who perceive these odours to be more pleasant are more likely to use perfumes.

Geranyl acetate was perceived to be more pleasant by Caucasians than by Asians (Figure 8e).

### Variability in odour quality perception

**Figure 9:**
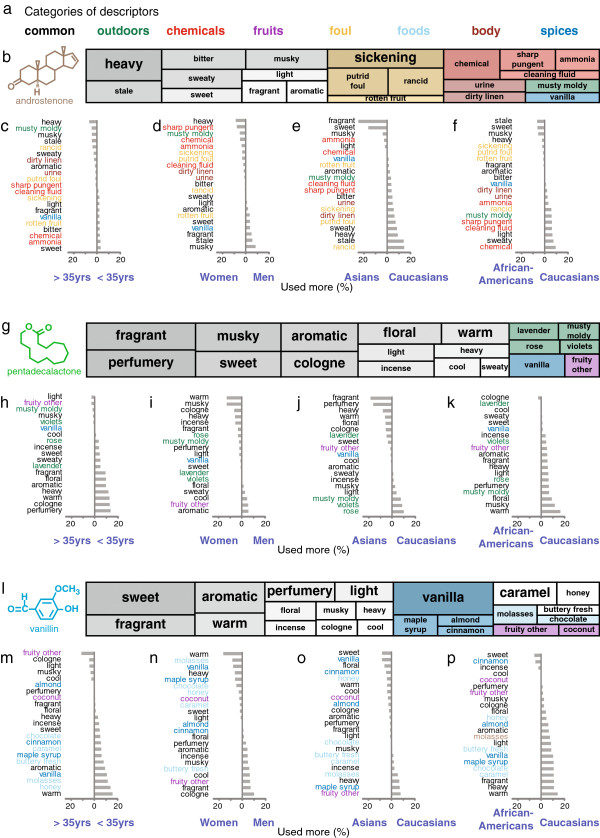
**Demographic differences in perceived odour quality.a**, Colour code for the categories of descriptors used. **b**, **g**, **l**, The molecular structure of androstenone (**b**), pentadecalactone (**g**), and vanillin (**l**) and the frequency with which descriptors were applied to each odour are shown. Only descriptors that were used in at least 10% of the visits are shown. The frequency of descriptor usage is represented by the area of the rectangles. **c**-**f**, **h**-**k**, **m**-**p**, The percentage differences between demographic groups in descriptor usage for descriptors that were applied to androstenone (1/10,000 dilution) (**c**-**f**), pentadecalactone (1/500 dilution) (**h**-**k**), or vanillin (1/200 dilution) (**m**-**p**) in more than 10% of the visits (Ns: < 35 years: 195; > 35 years: 196; Women: 210; Men: 181; Asians: 31; African-Americans: 97; Caucasians: 178). Subjects were divided by age according to the median of 34.6 years, which is rounded to 35 years for labeling the figure.

In addition to investigating the influence of demographic factors on intensity and pleasantness perception, we also assessed the perception of specific olfactory qualities. We tested olfactory qualities associated with three odours, androstenone, pentadecalactone, and vanillin, by asking subjects to assign descriptors from a list of 146 standard odour descriptors [65] (Figure 9). These 146 descriptors were grouped into eight categories and assigned a colour code (Figure 9a). After correcting for biases in descriptor usage by subtracting the descriptors used to describe the solvent, several differences in perceived odour quality emerged for each of the three odours tested. The descriptors applied to androstenone were largely based on the foulness of the smell (Figure 9b). As expected, pentadecalactone, a synthetic musk odour that is often sold under the brand name Exaltolide®, was described mainly with terms that can be applied to perfumes (Figure 9g), whereas for vanillin, perfume and food terms were used (Figure 9l). The differences between demographic groups in assigning descriptors to androstenone (Figure 9c-f), pentadecalactone (Figure 9h-k), and vanillin (Figure 9m-p) can uncover different associations or different perceptual qualities of the stimulus. Androstenone was more likely to smell “musky” and “aromatic” to women, whereas men found it to be more “chemical” and “sickening,” reflecting gender differences in the perceived qualities of the stimulus (Figure 9d).

## Conclusions

We have investigated the factors that influence olfactory perception in a large set of psychophysical data collected from a diverse population of subjects from the New York City metropolitan area. We found that within-individual variability did not differ more when tests were repeated months apart than when they were repeated minutes apart, suggesting that variability between tests is caused almost entirely by processes operating on a time-scale of minutes or seconds. We showed that general olfactory acuity correlates with age, gender, smoking habits, body type, and race. In addition we have identified over 100 cases in which sensitivity to a specific odour, or the intensity or pleasantness perception of a specific odour, differed significantly between demographic groups. We also studied in detail how the perceived odour quality of three odours differs between demographic groups.

Our results illustrate the complex composition of inter-individual variability in odour perception. If we for example consider the intensity rating of the high concentration of androstenone, which is the most variable of all the ratings reported here, we can assign multiple factors to the variability. One group of factors that contribute to this variability are factors that influence general olfactory function. Among those are genetic factors [8-10], which have been shown to explain about 20% of perceptual variability [66,67]. In addition there are environmental factors that contribute to the variability of olfactory acuity, such as prior upper respiratory infections, trauma, and environmental toxins [11,12,14]. The influence of environmental factors has been estimated to be larger than the influence of genetic factors [67]. However, the variability of the intensity perception of androstenone is not only caused by variability in general olfactory acuity. There are further factors that influence not all odour perception, but specifically the perception of androstenone and similar odours. Among these are environmental and genetic factors. The most important environmental factor that influences androstenone perception in an odour-specific fashion is prior exposure to the odour [46]. The most important genetic factor that has an odour-specific effect is probably variability in odorant receptor genes [68,69]. For the intensity perception of androstenone at high concentration in the subject population presented here, genetic variability in one of these receptors, OR7D4, has been shown to explain almost 40% of the perceptual variability [19].

The variability, subjectivity, or unreliability of olfactory perception is a major hurdle in understanding this enigmatic sense. Knowledge of the causes of perceptual variability will inform experimental designs in which the variability is controlled for. Recently, progress has been made in elucidating the genetic causes of inter-individual differences in the sense of smell [8-10,19-23]. Together with large psychophysical studies of demographic influences on smell perception like the one presented here, this research may one day make it possible to predict inter-individual differences in smell perception. Ultimately this approach has the potential to shed light on the innate and cultural factors that influence sensory perception and aesthetic preferences.

## Methods

### Subjects

Adult subjects were recruited from the New York City metropolitan area and tested between March 2005 and May 2006. All figures except Figure 3 are based on 391 subjects (210 women, 181 men). The median age of the subject was 34.6 years with a range from 19 to 75 years. For the analysis, subjects were divided into two groups, comprising the 196 older subject (“>35”) and the 195 younger subjects (“<35”). Of these 391 subjects, 91 were born outside the United States. 178 subjects self-identified as Caucasian, 97 as African-American, 31 as Asian, and 4 as Native American. There were no Pacific Islanders in this study. 70 subjects selected “Other” for self-reported race. Of these 70 subjects, 51 self-identified as Hispanics. Overall, 305 subjects self-identified as Non-Hispanics, 77 as Hispanics, 28 as underweight, 202 as of just the right weight, and 149 as overweight. During the first visit, the height and weight of 387 subjects were measured to calculate their body mass index (B.M.I.=mass), calculated as (kg)/[height (m)]^2^. We failed to obtain height and weight measures of 4 subjects. Subjects that self-identified as underweight had an average B.M.I. of 21.9±0.5, those that self-identified as of “just right” weight had a B.M.I. of 24.3±0.4, and those that self-identified as overweight had a B.M.I. of 30.8±0.6. The self-reported body weight data were well matched to typical boundaries used for clinical classifications of body weight. Subjects with a B.M.I. under 18.5 are considered underweight; a B.M.I. between 18.5 and 24.9 is considered normal weight; and a B.M.I. between 25 and 29.9 is considered overweight. People with a B.M.I. over 30 are considered to be obese. 290 subjects were non-smokers (defined as those who stated that they do not smoke or smoke very rarely; e.g. one cigarette per week). 92 subjects were smokers. 267 subjects used perfume at least once a week whereas 112 never used perfume. 366 of the subjects were from New York State, 19 from New Jersey, and 6 from elsewhere (Texas, Illinois, Pennsylvania, Florida, the United Kingdom) and on a short-term visit to the New York City area. Of the 366 subjects from New York State, 160 were from Manhattan, 75 from Brooklyn, 56 from Queens, 51 from the Bronx, 3 from Staten Island, and 21 from outside New York City. In some cases, demographic data do not add up to the number of subjects (391) because subjects were given the option not to answer any given demographic question. These missing data are indicated as “Do Not Wish To Specify” or “N/A” in the “demographics” tab of Additional file 1. All subjects gave their informed consent to participate and all procedures were approved by the Rockefeller University Institutional Review Board.

The data in Figure 3 are based on a subset of 56 subjects (35 women, 21 men; 28 Caucasians, 16 African-Americans, 12 Other) who were reinvited for a third and fourth visit more than one year after the first visit (Figure 2b). For Figure 3, we quantified within-individual variability by having these 56 subjects rate intensity and pleasantness of fifteen stimuli on eight occasions: twice within 30 minutes on four visits (Figure 2b). The first two visits were about one week apart and the third visit was scheduled more than one year later. Visit four was about one week after visit three.

All other figures are based on the data collected from 391 subjects (including the 56 subjects evaluated for Figure 3) who participated in the first two visits that were about one week apart. We attempted to eliminate the effects of within-individual variability by averaging the responses from these visits.

In total, 77% of enrolled subjects (N=412 subjects) completed the study, meaning that they completed two visits and provided a blood sample for genetic analysis. As in our previous analysis of these data [19], the 21 subjects (5%) with the lowest olfactory acuity were excluded from the analysis to avoid inclusion of malingerers and subjects with general anosmia. Methods to determine general olfactory acuity are described below.

### General psychophysics procedures

The psychophysical tests were self-administered and computerized using custom-written applications in FileMaker Pro and Microsoft Access. To ensure accuracy in data collection, all odour vials used in this study were barcoded. Barcoding had the further advantage that subjects were unaware what stimulus was contained in any given vial. Subjects scanned each odour vial containing the stimulus before opening the vial and were only permitted to proceed if the correct vial was scanned. During the first visit we collected data on the demographics, habits, and product usage of the subjects in a computer-administered questionnaire. Some of the results of the questionnaire are shown in Figure 1a-b.

### Intensity and valence rating

The intensity and valence of 66 odours at two concentrations (high and low) and two solvents (paraffin oil and propylene glycol) (Figure 2) were rated using a 7-point scale (Figures 3, 5d-h, 6, 7, 8). The odours tested (in alphabetical order) were: (−)-menthol, (+)-menthol, 1-butanol, 2-butanone, 2-decenal, 2-ethylfenchol, 2-methoxy-4-methylphenol, 4-methylvaleric acid, ambrette, androstadienone, androstenone, anise, banana, bourgeonal, butyl acetate, butyric acid, cedarwood oil, cineole, cinnamon, *cis*-3-hexen-1-ol, citral, citronella, decyl aldehyde, diacetyl, diallyl sulphide, diphenyl ether, ethyl vanillin, ethylene brassylate, eugenol, eugenol acetate, eugenol methyl ether, fenchone, fir, galaxolide, geranyl acetate, guaiacol, heptaldehyde, heptyl acetate, hexanoic acid, hexyl butyrate, isobornyl acetate, isobutyraldehyde, isobutyric acid, isoeugenol, isovaleric acid, jasmine, lime, linalool, methanethiol, methyl salicylate, nonyl aldehyde, nutmeg, octyl acetate, octyl aldehyde, orange, pentadecalactone, phenyl acetaldehyde, pyrazine, (*r*)-carvone, (*r*)-limonene, sandalwood oil, spearmint oil, terpineol, terpinyl acetate, undecanal, and vanillin. Odours were diluted in paraffin oil, except for (−)-menthol, (+)-menthol, androstadienone, androstenone, ethyl vanillin, pentadecalactone, pyrazine, and vanillin which were diluted in propylene glycol and methanethiol, which was diluted in water. Most odours were obtained from Sigma and of the highest purity available. The odour qualities that have been reported to be associated with these odours have been reported earlier [19]. Odour dilutions, solvent, and Chemical Abstracts Service (C.A.S.) numbers for all odours can be found in the “odours and sequence of stimuli” tab in Additional file 1.

For pleasantness, the rating scale was: “extremely unpleasant,” “very unpleasant,” “slightly unpleasant,” “neither unpleasant nor pleasant,” “slightly pleasant,” “very pleasant,” and “extremely pleasant.” For intensity, the rating scale was: “extremely weak,” “very weak,” “slightly weak,” “neither weak nor strong,” “slightly strong,” “very strong,” and “extremely strong.” In addition to the 7-point scale, there was a button on the screen labeled “I can’t smell anything” and a button labeled ”Don’t Know.“ If the ”Don’t Know” button was pressed, no rating was recorded. If the “I can’t smell anything” button was pressed, a 0 was recorded for the intensity rating and no rating was recorded for pleasantness.

Prior to these ratings, six stimuli that represented the spectrum of intensity and pleasantness of the stimuli used in the study were presented to allow the subjects to calibrate their usage of the scale (Figure 2c; grey ovals). These six calibration stimuli were terpineol (high); garlic (high); pyrazine (low); methanethiol (high); methyl salicylate (low), undecanal (high): (see the “odours and sequence of stimuli” tab in Additional file 1 for concentration and solvent information). The subjects were unaware that the first six stimuli served this purpose. After subjects had rated the solvents and 66 odours at two concentrations, 15 stimuli that were presented earlier in the experiment were repeated (Figure 2c; orange ovals). Odour stimuli were presented in the same order in all visits to facilitate comparisons between subjects. The complete sequence of all presented odours, their dilution, and solvent can be found in the “odours and sequence of stimuli” tab in Additional file 1.

To reduce olfactory adaptation or fatigue, the computer application for the intensity and valence rating was programmed to enforce a mandatory 15 second inter-stimulus interval. However, most subjects took longer than 15 seconds to move from one stimulus to the next, so this was rarely enforced. Although there was some variability between the first and second presentation of these stimuli, there was no indication of a systematic difference between the intensity rating at the beginning and end of the visit. Eight of the 15 stimuli were rated on average as more intense at the end of the visit, whereas seven were rated as less intense. This indicates that adaptation and olfactory fatigue during the testing did not systematically influence the results.

Prior to the study, the concentrations used for each odorant were determined in intensity-matching experiments in which subjects rated the intensity of stimuli. Odours were considered “low” intensity when the intensity rating was within one standard deviation of the intensity rating for an arbitrary low concentration odour standard, a 1:10,000 dilution of 1-butanol. Odours were considered “high” intensity when the intensity rating was within one standard deviation of an arbitrary high concentration odour standard, a 1:1,000 dilution of 1-butanol. For ethylene brassylate, eugenol methyl ether, (−)-menthol, (+)-menthol, and vanillin, the pure odour or the saturated dilution was rated less intense than the criteria for “high” intensity and these odours were therefore presented at the highest possible concentration. Androstenone and androstadienone could not be intensity matched in any meaningful way because of the high perceptual variability across subjects. Ten subjects participated in a pilot study aimed at intensity matching all stimuli and six visits for each subject were necessary to match all stimuli.

For the comparison between demographic groups in Figure 5d-h and Figure 8, the mean of the two visits was calculated for each subject. The stimuli were then ranked according to intensity (Figure 5d-h) or pleasantness (Figure 8) for each subject. The difference in mean rank of a stimulus between two demographic groups is shown.

### Detection thresholds

Detection thresholds (Figure 5a-c) were calculated using the single staircase method with seven reversals [70]. The thresholds were determined using a custom-built, computer-controlled, self-administered thresholding procedure.

Odour vials had barcode labels and the procedure was carried out at a computer equipped with a bar code scanner. Subjects were instructed to sniff two vials, one containing the solvent, the other a dilution of the odorant. Subjects were asked to scan the vial with the stronger odour. Depending on the answer, the procedure was repeated at an adjusted concentration.

### Assigning descriptors to odours

Subjects assessed the quality of androstenone, pentadecalactone, vanillin, and the solvent propylene glycol using a method that has been shown to produce stable profiles of odorants [59] (Figure 9). Subjects were asked to rate a list of 146 odour descriptors on a scale from 0 (“descriptor does not at all describe my perception of the odour”) to 5 (“descriptor perfectly describes my perception of the odour”), however, here it is only evaluated if a descriptor was applied to an odour at all or not. Descriptor assigning was performed as a computer-controlled self-administered experiment in which the subject’s responses were directly recorded. The default setting for each descriptor was set to 0, such that subjects recorded values from 1–5 for only those descriptors that pertained to their perception of a given odour. The data in Figure 9c-f, h-k, m-p are corrected for the descriptors used to describe the solvent (propylene glycol).

### Determining general olfactory acuity

We devised a measure of general olfactory acuity based on the data collected in this study. This measure of general olfactory acuity served two purposes. First, the 21 subjects (5%) with the lowest olfactory acuity were excluded from the analysis to avoid inclusion of malingerers and subjects with general anosmia. The prevalence of olfactory impairment in the United States is approximately 3.8% [35], so our exclusion criteria will exclude those suffering from damage to the olfactory system. Second, we used this measure to compare the olfactory acuity of demographic groups (Figure 4). Six performance indicators were ranked and the average of these six ranks was calculated as the general olfactory acuity, which is expressed as a rank from 1 (lowest acuity) to 391 (highest acuity). The six performance indicators were:

1. vanillin detection threshold

2. pentadecalactone detection threshold

3. isovaleric acid detection threshold

4. percentage of odours for which the “low” concentration was rated higher than the solvent

5. percentage of odours for which the “high” concentration was rated higher than the solvent

6. percentage of odours for which the “high” concentration was rated higher than the “low” concentration.

These six indicators are weakly correlated. The average Pearson’s correlation coefficient (r) between two of the indicators is 0.25. The notable exceptions are indicators 4 and 5, which are both strongly influenced by how the subjects rate the intensity of the solvent and are therefore strongly correlated (r=0.81). Despite this weak correlation between the indicators, the resulting measure of general olfactory acuity is stable. If the olfactory acuity is calculated using only five of the six indicators, the average Pearson’s correlation coefficients between the resulting six measures that are based on five indicators is 0.93. This shows that no single indicator contributes disproportionally to the measure of general olfactory acuity used here.

### Data sharing

The analysis presented here highlights some interesting observations that resulted from mining a large psychophysical dataset. Many additional questions can be addressed using this dataset. The analysis presented here can be refined, for example by combining demographic groups and comparing, for example, male perfume-users and female perfume-users. But these data will also be useful in addressing questions unrelated to perceptual variability. There are for example strong correlations between many of the different perceptual measures employed here and between those measures and structural features of the molecules. To enable the scientific community to perform further analysis with this dataset, we include the raw data as Additional file 1.

## Abbreviations

C.A.S.: Chemical Abstracts Service; B.M.I.: Body mass index; S.D.: Standard deviation of the mean.

## Competing interests

The authors declare a competing interest. LBV is a member of the scientific advisory board of International Flavors & Fragrances, Inc. and receives compensation for these activities.

## Authors’ contributions

AK carried out all the experiments and analysed the data. MH and IAG carried out the psychophysical testing and ANG contributed to psychophysical study design. AK and LBV together designed the experiments, interpreted the results, produced the figures, and wrote the paper. All authors read and approved the final manuscript.

## Supplementary Material

Additional file 1 Perceptual data. Click here for file
